# Ligation-assisted endoscopic full-thickness resection combined with preloaded sutures for tiny mesenchymal tumors of the gastric fundus

**DOI:** 10.1055/a-2337-7647

**Published:** 2024-07-03

**Authors:** Minna Zhang, Peng Shen, Wenzhuo Zhao, Weijie Dai, Xiaozhong Yang, Rui Xie

**Affiliations:** 1 Department of Gastroenterology, The Affiliated Huai’an No.1 People’s Hospital of Nanjing Medical University, Huaian, China

Gastrointestinal stromal tumors (GISTs) are the most prevalent tumors of mesenchymal tissue origin in the gastrointestinal tract 11 . Currently, the treatment of small GISTs (≤2 cm) and micro-GISTs (<1 cm) remains controversial. Endoscopic full-thickness resection (EFTR) is indicated for microscopic GISTs originating from the intrinsic muscularis propria, facilitating thorough tumor removal and minimizing the risk of dissemination 22 . The fundus of the stomach is one of the commoner sites for GISTs, and performing EFTR here requires high levels of endoscopic skill and, because of the small size of the tumor, it is very easy for it to fall into the abdominal cavity after the final resection 33 . To overcome this challenge, we used a transparent cap-assisted endoscopic full-thickness ligation (EFTR-L) technique combined with preloaded sutures ( Video 1Video 1 ), which allowed not only complete tumor resection and rapid specimen recovery, but also the prevention of intraoperative bleeding and perforation by use of the preloaded sutures.

A transparent cap-assisted endoscopic full-thickness ligation technique combined with preloaded sutures is used to resect a tiny mesenchymal tumor in the gastric fundus.Video 1Video 1

A 45-year-old man was found to have a 0.7-cm hemispherical bulge on the fundus of the stomach during gastroscopy ( Fig. 1Fig. 1 **a** ). Endoscopic ultrasound suggested that the lesion was a hypoechoic mass of submucosal intrinsic muscular layer origin in the gastric fundus ( Fig. 1Fig. 1 **b** ). With the EFTR-L approach, we first drew the lesion into the lancing cap with forceful suction ( Fig. 2Fig. 2 **a** ). Localized ligation of the lesion was performed using a lancing device to form a pseudo-polypoid bulge ( Fig. 2Fig. 2 **b** ). The ligature ring was then removed and three metal clips were pre-positioned around the tumor with nylon cords to form the shape of a purse-string suture ( Fig. 2Fig. 2 **c** ). Next, the root of the tumor was encircled using a loop device, which was gradually tightened and lifted, while the nylon cord was tightened to pre-close the peripheral tissues of the lesion, before the mass was excised in its entirety ( Fig. 2Fig. 2 **d** ). Ultimately, the gastric fundus mass was swiftly and entirely excised with no post-procedural bleeding or exposure of muscular tissue ( Fig. 2Fig. 2 **e, f** ).

**Fig. 1:**
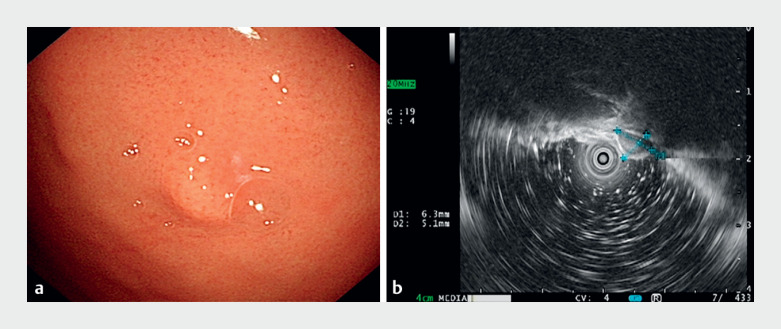
**Fig. 1** A submucosal mass in the fundus of the stomach is seen on: **a** endoscopic view, where it presents as a submucosal bulge with a smooth surface; **b** endoscopic ultrasound, which shows that the lesion originates in the lamina propria, is hypoechoic, has an intact peritoneum, and measures approximately 6.3 × 4.8 mm.

**Fig. 2:**
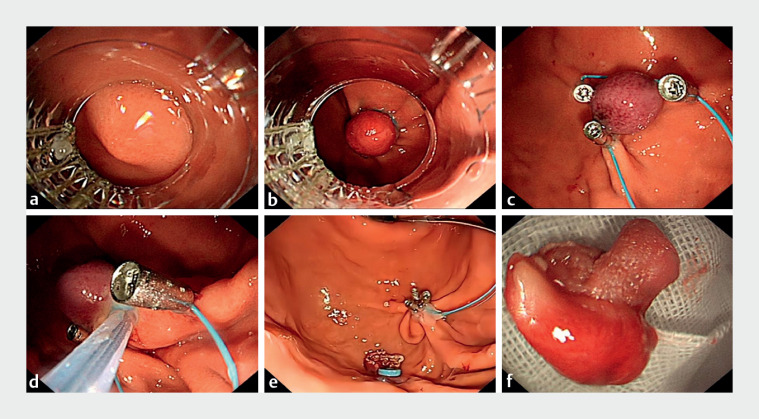
**Fig. 2** Endoscopic images of the treatment process showing: **a** after installation of the ligature at the end of the endoscope, the mirror being used to find the tumor, adjust the angle, and slowly draw the tumor into the ligature; **b** a pseudo-polypoid bulge that is formed after correctly aligning the head end of the ligature, applying negative pressure to draw the tumor completely into the transparent cap, and releasing the ligature ring; **c** three metal clips placed around the tumor after removal of the ligature ring, with a nylon rope forming a purse-string suture; **d** gradual tightening of the loop ligature ring and lifting around the root of the tumor, which is performed simultaneously with tightening of the nylon cord to pre-close the peripheral tissues of the lesion, before resection of the entire lesion; **e** the final appearance of the tightened purse-string suture after tumor resection. **f** The macroscopic appearance of the resected specimen.

This approach not only ensures the effectiveness and safety of the procedure, but also reduces both the duration of the procedure and the post-procedure hospitalization, rendering it innovative and worthy of clinical promotion.

Endoscopy_UCTN_Code_TTT_1AO_2AG_3AF

## Notes

Endoscopy E-Videos https://eref.thieme.de/e-videos
*E-Videos* is an open access online section of the journal *Endoscopy* , reporting on interesting cases and new techniques in gastroenterological endoscopy. All papers include a high-quality video and are published with a Creative Commons CC-BY license. Endoscopy E-Videos qualify for HINARI discounts and waivers and eligibility is automatically checked during the submission process. We grant 100% waivers to articles whose corresponding authors are based in Group A countries and 50% waivers to those who are based in Group B countries as classified by Research4Life (see: https://www.research4life.org/access/eligibility/ ). This section has its own submission website at https://mc.manuscriptcentral.com/e-videos .
